# Identification of a novel mutation in the *PNLIP* gene in two brothers with congenital pancreatic lipase deficiency

**DOI:** 10.1194/jlr.P041103

**Published:** 2014-02

**Authors:** Doron M. Behar, Lina Basel-Vanagaite, Fabian Glaser, Marielle Kaplan, Shay Tzur, Nurit Magal, Tal Eidlitz-Markus, Yishay Haimi-Cohen, Galit Sarig, Concetta Bormans, Mordechai Shohat, Avraham Zeharia

**Affiliations:** * Raphael Recanati Genetics Institute, Schneider Children's Medical Center of Israel, Rabin Medical Center, Petah Tikva, Israel; † Felsenstein Medical Research Center, Schneider Children's Medical Center of Israel, Rabin Medical Center, Petah Tikva, Israel; § Beilinson Campus, and Pediatric Genetics, Schneider Children's Medical Center of Israel, Rabin Medical Center, Petah Tikva, Israel; ** Day Hospitalization Unit, Schneider Children's Medical Center of Israel, Rabin Medical Center, Petah Tikva, Israel; †† Molecular Medicine Laboratory, Rambam Medical Center, Haifa, Israel; §§ Laboratory of Clinical Biochemistry, Rambam Medical Center, Haifa, Israel; *** Hematology Laboratory, Rambam Medical Center, Haifa, Israel; ††† The Lorry I. Lokey Interdisciplinary Center for Life Sciences and Engineering, Technion - Israel Institute of Technology, Haifa, Israel; §§§ Sackler Faculty of Medicine, Tel Aviv University, Tel Aviv, Israel; **** Genetics and Genomic Medicine Laboratory, DNATraits, GenebyGene, Houston, TX

**Keywords:** dietary fat, absorption, consanguineous, Arab

## Abstract

Congenital pancreatic lipase (PNLIP) deficiency is a rare monoenzymatic form of exocrine pancreatic failure characterized by decreased absorption of dietary fat and greasy voluminous stools, but apparent normal development and an overall good state of health. While considered to be an autosomal recessive state affecting a few dozens of individuals world-wide and involving the *PNLIP* gene, no causative mutations for this phenotype were so far reported. Here, we report the identification of the homozygote missense mutation, Thr221Met [c.662C>T], in two brothers from a consanguineous family of Arab ancestry. The observed genotypes among the family members were concordant with an autosomal recessive mode of inheritance but moreover a clear segregation between the genotype state and the serum PNLIP activity was evident. Based on biophysical computational tools, we suggest the mutation disrupts the protein's stability and impairs its normal function. Although the role of PNLIP is well established, our observations provide genetic evidence that *PNLIP* mutations are causative for this phenotype.

In triglyceride form, lipids cannot be absorbed (1, 2). The three main factors which are involved in the duodenal digestion of dietary triglycerides in mammals include two proteins, pancreatic lipase (PNLIP) and colipase (CLPS), and bile salts (3). Human PNLIP is a 56 kDa protein secreted by the acinar pancreas and is essential for the hydrolysis and absorption of long-chain triglyceride fatty acids in the intestine (4). In vivo, the 12 kDa protein cofactor, CLPS (5), is required to anchor PNLIP to the surface of lipid micelles, counteracting the destabilizing influence of bile salts (2, 3). The Online Mendelian Inheritance in Man phenotype 614338, including congenital PNLIP deficiency, congenital pancreatic CLPS deficiency, and combined congenital PNLIP and CLPS deficiency, is a rare monoenzymatic form of exocrine pancreatic failure. No more than a few dozens of patients have been described so far in the literature with a variable age of onset ranging from infancy to adulthood (6–16). The clinical synopsis includes greasy and voluminous stools coupled with evidence of decreased absorption of dietary fat and isolated or combined PNLIP and CLPS deficiencies. Remarkably, despite these findings patients’ growth and state of health are not compromised. While the inheritance mode of the condition is believed to be autosomal recessive, no causative mutations have been documented thus far. We report the genetic investigation and resolution of a PNLIP deficiency state in two brothers presenting the classical signs and symptoms of this rare phenotype.

## METHODS

### Subjects

**Fig. 1.**
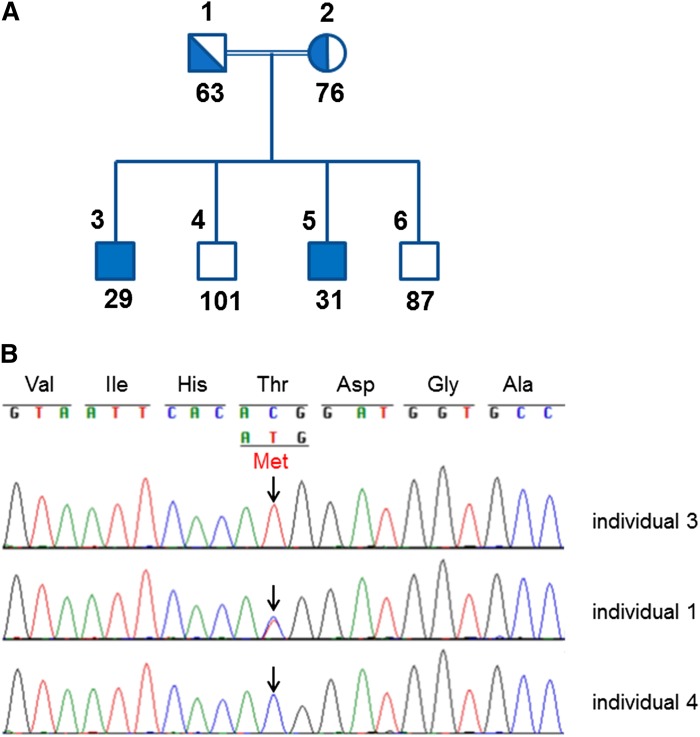
Identification of the *PNLIP* gene, c.662C>T, p.Thr221Met mutation by sequence analysis. A: Family members are marked using the digits 1–6 as noted above the pedigree symbols. PNLIP activity (U/l) for each family member is shown below the symbols. B: Sequences from one homozygote affected sibling (individual 3), one heterozygote carrier parent (individual 1), and one homozygote WT noncarrier sibling (individual 4) are shown. Position c.662 is marked by the arrows.

The studied family comprised six family members including two parents, two affected, and two unaffected siblings (**Fig. 1**). The parents are first degree cousins and the family is of Arab Muslim ancestry. In addition, a set of 150 samples (300 chromosomes) of Arab Muslims from central Israel available at our laboratory was used to calculate the carrier frequency. The institutional review board at the Rabin Medical Center approved the study. Written informed consent was obtained from all participants or from their parents.

### Biochemical measurements

Laboratory work-up including complete cell count, chemistry, coagulation, lipidogram, and direct measurements of vitamins A, D (25 OH vitamin D3), and E was carried out for all family members. Coagulation tests performed as indirect indicators of vitamin K deficiency included prothrombin time (PT)/partial thromboplastin time (PTT)/international normalized ratio. Serum vitamin E and vitamin A levels were determined by reverse phase high-performance liquid chromatography with UV spectrophotometric detection (UV JASCO 2075 PLUS Detector, USA). Serum samples were deproteinized with ethanol and extracted once with n-hexane. Resulting extracts were injected onto a C18 reversed phase column eluted with 100% methanol. The detection was operated using a channel of a diode-array spectrophotometer at 290 nm. The internal standard used was α-tocopherol acetate (17). Serum vitamin D levels were detected using the DiaSorin LIAISON 25 OH vitamin D total assay, which is a direct competitive chemiluminescence immunoassay for quantitative determination of total 25 OH vitamin D in serum. Total serum PNLIP activity, which reflects mainly PNLIP activity, was determined by colorimetry using commercial reagents (DF56, Siemens, Germany). The assay was performed on an automated chemistry analyzer (Dimension RXL, Siemens).

### Molecular analysis

Genomic DNA was extracted from peripheral leukocytes following standard protocols. DNA was amplified to obtain all *PNLIP* (4) and *CLPS* (5) genes coding exons and their flanking regions using conventional PCR techniques. PCR products were purified using magnetic particle technology (Seradyn, Inc.). After purification, all fragments were sequenced by forward and backward internal primers to determine the noted regions. The primers used for PCR of the *PNLIP* exons 1–12 are as follows: 1F, TATCCATTTTCCCCCAGAGG; 1R, GAATTCAGCTGCCCTAGCC (215 bp); 2F, GTGGCTAGGAGGGTGTTGAG; 2R, ATCTCCTGACCTCGTGAACC (359 bp); 3F: TCTTCTACTTACTGCCCCTCTCC; 3R, GCCAGTGCCTTACCGTTTG (280 bp); 4F, TGACATTGTGTGGGTCAAGG; 4R, TTCTTTGTAGCCAAGAGAAACATC (388 bp); 5F, TTCCCACAACAATGAAATCG; 5R, AGATTGGCCTCAAACTGCTG (300 bp); 6 and 7F, TCATCCCTTTCCATGCATAAC; 6 and 7R, TCCAAAGAATTTAGCAAGTTTAGC (500 bp); 8F, GTTCTGTGACCTGCATGAGC; 8R, TCATGTATAGGAAAGCACACCAG (319 bp); 9F, GCGACAACATGTAGGAAATATGG; 9R, GCAAGTATGCACATTTTCAGTG (268 bp); 10F, TCAAAACGTGGCAGTAGTGG; 10R, AGCTACTCGGGAGGCTAAGG (385 bp); 11F, AGAAATGCATTGTAAGCTGGTC; 11R, TCCCCTGTAGGAGGTTTCAC (399 bp); 12F, GGGGCATAGATTGTCCTCAC; 12R, TTGAAACAATCGAGGTTTTGG (244 bp). PCR was performed using the M13-tagged primers M13F TGTAAAACGACGGCCAGT and M13R CAGGAAACAGCTATGACC, using the following cycling conditions: 94°C 10 min × 1 cycle; 94°C 20 s; 58°C 20 s; 72°C 2 min; 40 cycles; 72°C 8 min × 1 cycle; 4°C hold. Sequencing was performed using M13 forward and reverse primers under the following conditions: 96°C 1 min × 1 cycle; 96°C 10 s; 50°C 5 s; 60°C 2 min; 45 cycles; 4°C hold. Sequencing was completed on a 3730xl DNA analyzer (Applied Biosystems), and the resulting sequences were analyzed with the Sequencher software (Gene Codes Corporation). Mutations were scored relative to the reference sequences deposited in the National Center for Biotechnology Information [*PNLIP* (NM_000936), *CLPS* (NM_001832 and NM_001252597)].

## RESULTS

### Description of study participants

Two brothers, 15 and 19 years old, were referred for genetic counseling due to a history of greasy and voluminous stools since birth and a suggested diagnosis of PNLIP deficiency. The brothers are the first and third of four brothers born to healthy first cousin consanguineous parents of Arab Muslim origin (Fig. 1). The elder brother was first seen at our day hospitalization unit at the age of two years. Obstetric history and developmental milestones were unremarkable. The parents described greasy stools since the first postnatal days of feeding. Prior investigation yielded no clinical diagnosis. On physical examination he had no dysmorphic features or physical abnormalities. Repeated height and weight measurements until adulthood remained in the 90th and 75th–90th percentile, respectively. An extensive laboratory analysis commenced. Cell blood count and coagulations (PT/PTT) tests were normal. A lipase level of 6 U/l (16–63 U/l) was noted. Normal cholesterol and triglyceride levels of 133 mg/dl and 89 mg/dl, respectively, were measured. A low β-carotene level of 25 μg/dl (50–250 μg/dl) and a low vitamin E level of 0.4 mg/dl (0.5–2 mg/dl) were measured. The vitamin D level was normal. Serum iron, vitamin B12, and folic acid values were normal. The xylose test was normal. The sweat test was normal. Alpha-1-antitrypsin was not detected in the stool and there were no indications for a protein-losing enteropathy. A fecal elastase level of 23 μg/g stool was recorded (>200 μg/g stool). The pancreolauryl test was abnormal. A 3 day stool collection yielded 82 g of fat indicating fat malabsorption. The small intestine biopsy was normal. Following this work-up the diagnosis of congenital PNLIP deficiency was proposed but was not proved genetically. ADEK vitamin replacements and Creon were administered. The younger brother was first seen in our department at the age of two years. The parents explained that they recognized the phenotype in the first postnatal days but because they were aware of its benign nature did not seek medical advice. On physical examination he had no dysmorphic features or physical abnormalities. Repeated height and weight measurements until adulthood remained in the 75th and 75th–90th percentile, respectively. Laboratory analysis demonstrated normal PT/PTT and vitamin D levels. A lipase level of 7 U/l (16–63 U/l) was noted. A low β-carotene level of 29 μg/dl (40–220 μg/dl) and a low vitamin E level of 0.3 mg/dl (0.5–2 mg/dl) were measured. A fecal elastase level of 43 μg/g stool was recorded (>200 μg/g stool). Alpha-1-antitrypsin was not detected in the stool and there were no indications for a protein-losing enteropathy. ADEK vitamin replacements and Creon were administrated.

At the genetic counseling that led to the identification of the deleterious *PNLIP* gene mutation reported herein, the brothers described greasy and voluminous stools following meals but reported favorably on their fitness and “inability to gain weight despite very high caloric intake,” which distinguished them from their other family members. They were both treated with ADEK vitamin replacements and Creon since childhood with partial compliance. The brothers considered themselves to be healthy and reported no disabilities other than the inconvenience related to the greasy and voluminous stools. Their physical examination was normal. The height and weight of the elder brother were 183 cm and 74 kg (BMI = 22), respectively. The height and weight of the younger brother were 160 cm and 60 kg (BMI = 23.4), respectively. The family requested prenatal counseling as part of family planning of the elder brother. Following written informed consent, a genetic work-up was initiated on all family members.

### Biochemical analysis

PNLIP activity (Fig. 1, **Table 1**), measured in all family members, demonstrated a dramatic decrease in the measured levels for the two affected brothers, 29 and 31 U/l (73–393 U/l), respectively, while normal levels of 101 and 87 U/l were noted in the two unaffected brothers. The healthy father showed a mildly reduced PNLIP activity of 63 U/l and the healthy mother showed a low value of 76 U/l, within the normal range of PNLIP activity.

**TABLE 1.** Fasting PNLIP activity; vitamins A, D, and E; PT/PTT; and lipid profile of all family members

Pedigree	Age (years)	PNLIP*^a^* (73– 393 U/l)	VIT-A*^b^* (μg/dl)	VIT-D*^c^* (ng/ml)	VIT-E*^d^* (mg/dl)	PT/PTT	TC (120–200 mg/dl)	HDLs (30–65 mg/dl)	TGs (30–170 mg/dl)	LDLs (75–140 mg/dl)	ApoA1 (1.1–2.2 g/l)	ApoB (0.55–1.35 g/l)
1 (c.662C>T/+)	43	63	72.5	21	1.16	Normal	189	34	143	126	1.34	1.02
2 (c.662C>T/+)	38	76	67.5	4.46	0.82	Normal	161	39	66	109	1.15	0.5
3 (c.662C>T/c.662C>T)	19	29	46.7	18	0.54	Normal	117	49	59	56	1.28	0.4
4 (+/+)	17	101	68	23.3	0.67	Normal	125	42	40	75	1.16	0.43
5 (c.662C>T/c.662C>T)	15	31	39	17.8	0.49	Normal	112	34	36	71	1.09	0.62
6 (+/+)	3	87	32.2	20.1	0.93	Normal	142	45	30	91	1.37	0.64

VIT-A, vitamin A; VIT-D, vitamin D; VIT-E, vitamin E; TC, total cholesterol.

a PNLIP activity.

b Vitamin A, normal ranges are age dependent and as follows: 0–6 years, 20–43 μg/dl; 7–12 years, 26–49 μg/dl; 13–17 years, 26–72 μg/dl; ≥18 years, 30–80 μg/dl.

c Vitamin D, ranges are defined as sufficient >30 ng/ml; insufficient 20–30 ng/ml; deficient 10–20 ng/ml; severely deficient <10 ng/ml.

d Vitamin E, normal ranges are age dependent and as follows: 0–18 years, 0.38–1.84 mg/dl; ≥18 years, 0.55–1.70 mg/dl.

Vitamin A, D, and E values were measured in all family members (Table 1). It is important to reiterate that the two affected brothers were treated with ADEK vitamin supplements at the time of the chemical analysis. The healthy mother showed a severely deficient vitamin D level of 4.5 ng/ml (severely deficient <10 ng/ml). The eldest of the two affected brothers showed a vitamin E level of 0.54 (0.55–1.70 mg/dl), which marks the lower range of the normal values for his age group. While the vitamin E level measured for the second affected brother and the vitamin A levels measured for the two affected brothers seemed to be lower than the values observed for the other family members, they were still within the normal ranges.

The blood lipid profiles of all family members are shown in Table 1. The two affected brothers show slightly decreased total cholesterol levels of 117 and 112 mg/dl. Complete cell counts, chemistry, and coagulation tests were normal for all family members.

### Mutation identification

A homozygous single base substitution in exon 6 of the *PNLIP* gene (g.118314780C>T; c.662C>T), resulting in a missense mutation at amino acid position 221 (p.Thr221Met), was detected in both probands (Fig. 1). The parents were heterozygous for the observed mutation and the two unaffected brothers were homozygous for the WT allele. Thus, a perfect segregation between the genotype state and the serum PNLIP activity was evident. No mutations were found within the boundaries of the coding exons and the splicing regions of the *CLPS* gene. The *PNLIP* gene c.662C>T mutation was not previously reported in publicly available databases and is predicted to be deleterious by PolyPhen2 (HumDiv and HumVar) (18), SIFT (19), and Mutation Taster (20). We screened for the mutation in a set of 150 samples (300 chromosomes) of Arab Muslims from central Israel available at our laboratory and found a total of 0 chromosomes carrying this mutation, indicating a 95% confidence interval of 0–1.2% for carrier frequency in this population using the Poisson exact confidence interval approach.

### Prediction of mutation impact on PNLIP function and structure

**Fig. 2.**
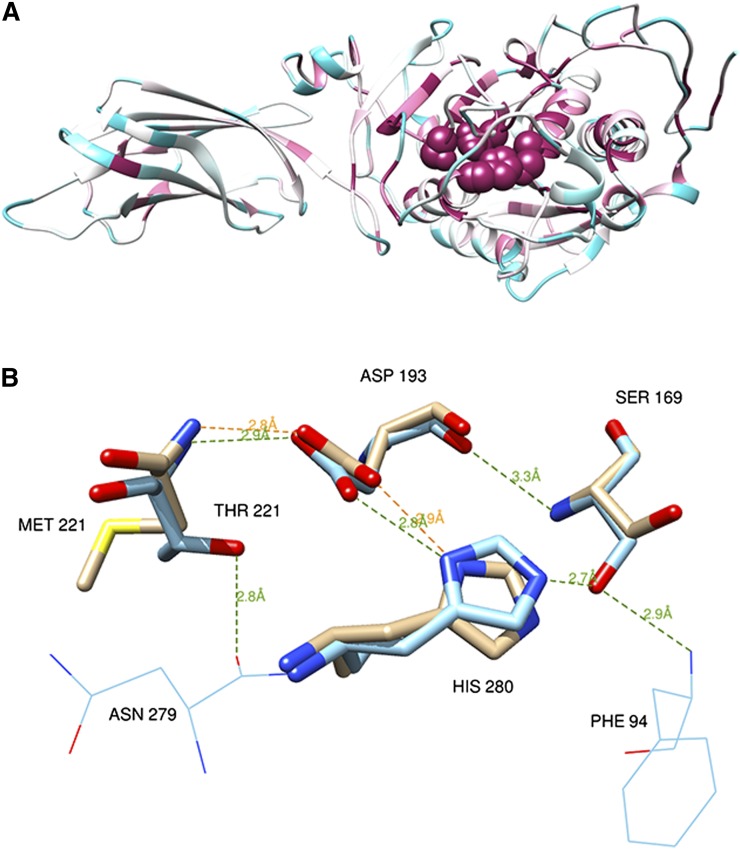
PNLIP protein structure, PDB 1n8s, chain A. A: PNLIP protein fold color-coded by evolutionary conservation (variable to conserved residues colored from cyan to maroon) as obtained from the ConSurf server (35). Active site catalytic residues Ser169, His280, Asp193, and Thr221 (shown as spheres) are highly conserved residues located on the heart of the Rossman fold scaffold domain. B: Hydrogen bonds of the catalytic site residues and mutation site on the WT structure (carbons colored cyan) and the Thr221Met mutant (carbons colored tan). While the WT structure in the vicinity of the catalytic site is involved in at least six hydrogen bonds (green dashed lines), only two of them are conserved in the modeled mutant structure (orange dashed lines). The figures and hydrogen bond calculations were produced by Chimera (36). Molecular graphics and analyses were performed with the University of California San Francisco Chimera package.

PNLIP (PDB ID: 1n8s) contains a catalytic triad structure buried under a lid cover (2). The triad encompasses amino acids Ser169, His280, and Asp193 (21), and it is located on the Nterm alpha-beta Rossman fold domain. Similar triad structures are found in a wide range of enzymes capable of hydrolysis of various substrates. The catalytic triad and Thr221 site are located in an ultra-conserved region, indicating their functional and structural critical importance for the protein activity and fold (see **Fig. 2A**). Analysis of the catalytic site structure of the PNLIP protein (22) (Fig. 2B) indicates that the mutation site p.Thr221Met reported herein is located very close to the PNLIP catalytic triad and supports its stability by two hydrogen bonds, one with Asp193 and one between the side chain oxygen of Thr221 and the backbone oxygen of Asp279. On the Thr221Met mutant the small polar amino acid threonine is replaced by the larger nonpolar amino acid methionine. In order to test the structural and functional impacts of the Thr221Met mutations, we used the Eris server (23), which calculates the change of the protein stability induced by mutations utilizing the Medusa atomic-modeling suite. Furthermore, Eris models backbone flexibility, which turns out to be crucial for ΔΔG estimation of small-to-large mutations, as in the case of Thr221Met. The Eris server calculated a ΔΔG of 8.28 kcal/mol for the PNLIP Thr221Met mutation, which strongly suggests that this mutation induces a serious destabilization of the protein structure compared with the WT. The comparison between the mutant and the WT structures, including a region of radius 5 Å around the catalytic residues (i.e., 31 residues), showed a significant root mean square deviation of 0.291 Å. This structural destabilization caused, in turn, the loss of several hydrogen bonds (Fig. 2B) that kept Ser169 and His280 tightly in place in the WT, and therefore a direct and important impact on the catalytic function could be expected to occur. The Thr221Met mutation was then predicted to cause a structural destabilization of the highly conserved catalytic binding machinery, which could be the cause of the observed dramatic decrease in PNLIP activity.

## DISCUSSION

To our knowledge, this is the first report identifying a *PNLIP* gene mutation as the cause of congenital PNLIP deficiency. Fat-soluble vitamins were specifically measured, but the interpretation of the results was complicated due to the ADEK supplementation given to the probands. Concordant with previous reports, the probands demonstrated low levels of vitamins A and E. Though vitamin D levels of the two affected brothers were slightly below the cutoff for deficient, clinical vitamin D measurements are not adequately precise to distinguish these values from the values measured for their father and the two unaffected brothers. The vitamin D level measured for the mother was severely deficient, which is likely the result of other factors (24). PNLIP activity of the two affected brothers was remarkably decreased while the two unaffected brothers and the parents showed normal and intermediate results, respectively (Table 1). These results indicate that heterozygotes have a lower enzyme activity level which seems to be sufficient to enable normal function and thus prevent phenotypic expression. Table 1 also denotes the lipid profiles of all family members. Interestingly, the two affected brothers demonstrated slightly decreased levels of total cholesterol, which can be considered to be at the range of hypolipidemia (total cholesterol < 120 mg/dl). While the clinical synopsis of the PNLIP deficiency phenotype does not include hypolipidemia, it was previously shown that pancreatic insufficiency is one of the causes for secondary hypolipidemia (25). Importantly, previous reports demonstrated that even with a nearly complete absence of PNLIP or CLPS, fat absorption was ∼70% of fat intake indicating extrapancreatic sources of lipase (11–13, 15, 16). Accordingly, lingual (26), gastric (2, 27,) and the bile salt-stimulated lipase (28) might have a compensating role preventing the severe manifestation of fat malabsorption and ADEK vitamin deficiencies.

The reported mutation should be considered for testing in patients presenting the classical signs and symptoms of congenital PNLIP deficiency among Arab Muslims primarily from central Israel. Despite our carrier screening estimates, precise knowledge of the carrier frequency remains controversial. While low in the general Arab Muslim population screened herein, the practice of consanguinity among Arab Muslims in Israel (29, 30) might result in much higher carrier frequencies of the mutation in the specific village and kindred of the probands.

While the potentially devastating clinical outcomes of prolonged vitamin deficiencies and the clear inconveniences involved with the phenotype of voluminous and greasy stools mandates monitoring and extensive administration of ADEK vitamins and pancreatic extract replacement, the long-term outcome and potential protective effect of lower fat absorption on the risk of developing obesity warrants discussion. Obesity, defined as excessive fat accumulation, was recognized by the World Health Organization to have more than doubled since 1980 worldwide (31). Obesity is associated with diabetes, hypertension, coronary heart disease, stroke, hyperlipidemia, osteoarthritis, several types of cancer, gallbladder disease, nonalcoholic steatohepatitis, sleep apnea, infertility, and depression. Some of these diseases are the main cause of mortality in developed countries, which is the reason for the continuous search for efficient treatment of obesity, one of which is the use of anti-obesity drugs (32). Much in line with our report, the only anti-obesity drug currently approved in Europe is Orlistat, a PNLIP inhibitor (33). Orlistat was approved by the US Food and Drug Administration in 1999 and for over-the-counter sales in 2007. The European Medicines Agency approved Orlistat in 1998. Orlistat is the saturated derivative of lipstatin, a potent natural irreversible inhibitor of PNLIP isolated from the bacterium *Streptomyces toxytricini*. Orlistat blocks the absorption of up to one third of ingested fat with minimal systemic absorption. The XENDOS study concluded that Orlistat therapy reduced not only weight, but also the incidence of diabetes beyond the result achieved with lifestyle changes (34). Not surprisingly, the most common side effects are diarrhea and steatorrhea. As the research for anti-obesity drugs is ongoing, it is possible that better knowledge about the types and locations of causative congenital deleterious *PNLIP* gene mutations might aid in designing and targeting critical domains of PNLIP. Accordingly, the exact effect of the herein described N-terminal mutation p.Thr221Met on the tertiary structural and functionality of PNLIP remains the scope of future studies.
